# BASP1 Suppresses Cell Growth and Metastasis through Inhibiting Wnt/*β*-Catenin Pathway in Gastric Cancer

**DOI:** 10.1155/2020/8628695

**Published:** 2020-12-24

**Authors:** Li Li, Qinghua Meng, Guoying Li, Limei Zhao

**Affiliations:** ^1^Department of Spleen-stomach hepatobiliary, Jinan Zhangqiu District Hospital of TCM, Ji'nan, Shandong 250200, China; ^2^Endoscopy Center, YuCheng People's Hospital, Yucheng, Shandong 251200, China

## Abstract

**Objective:**

Our research is designed to explore the function of brain acid soluble protein 1 (BASP1) in the progression of gastric cancer (GC) and its underlying molecular mechanisms.

**Methods:**

In this study, the expression of BASP1 was detected by quantitative real-time polymerase chain reaction (qRT-PCR) in both GC tissue and GC cells. The cell cloning, proliferation, apoptosis, migration, and invasion potential of AGS and HGC-27 cells were, respectively, determined using colony formation assay, 5-ethynyl-20-deoxyuridine (EDU) assay, flow cytometry, and Transwell assay. The protein expressions of Bax, caspase-3, Bcl-2, matrix metalloproteinases 2 (MMP-2), MMP-9, Wilms tumor 1 (WT1), Wnt, and *β*-catenin in AGS and HGC-27 cells were measured by western blot. In addition, the mRNA expressions of WT1, Wnt, and *β*-catenin in AGS and HGC-27 cells were detected by qRT-PCR.

**Results:**

BASP1 expression was significantly downregulated in both GC tissue and GC cells. BASP1 overexpression markedly repressed proliferation, migration, and invasion and facilitated apoptosis in AGS and HGC-27 cells. In addition, BASP1 overexpression notably promoted the protein expression of Bax and caspase-3 in AGS and HGC-27 cells and inhibited the expression of Bcl-2, MMP-2, and MMP-9. Moreover, BASP1 overexpression significantly inhibited the mRNA and protein expression of WT1, Wnt, and *β*-catenin in AGS and HGC-27 cells.

**Conclusion:**

BASP1 could significantly suppress cell proliferation, migration, and invasion and promote apoptosis through inhibiting the activation of the Wnt/*β*-catenin pathway in GC.

## 1. Introduction

Gastric cancer (GC) is the fifth most common cancer and the third-highest cause of death related to cancers worldwide [1, 2]. Over the past few decades, despite substantial improvements made in the treatment of GC patients, tumor recurrence and metastasis are still the leading causes of death in patients with GC [3, 4]. Hence, it is vital to explore novel prognostic markers and therapeutic targets for the treatment of GC.

Brain acid soluble protein 1 (BASP1) was initially identified as an abundant membrane-bound protein in the brain [5]. More and more studies have reported that BASP1 could regulate various types of cell biological behaviors including proliferation, apoptosis, and differentiation [6–8]. Increasing evidence has confirmed that BASP1, a potential tumor suppressor, plays an important role in a variety of cancers, such as thyroid cancer [5], liver cancer [9], and lung cancer [10]. However, the effect of BASP1 on GC has not yet been reported.

BASP1 is reported to be a component of Wilms tumor 1 (WT1) cosuppressor which can encode transcription factors [11]. Studies have indicated that WT1 can facilitate tumor progression as an oncogene through modulating tumor cell proliferation, apoptosis, and metastasis [12]. WT1 has been shown to activate the Wnt/*β*-catenin pathway in multiple cancers [10]. Previous studies have confirmed that the Wnt/*β*-catenin pathway plays a key role in the process of multiple human malignancies by regulating cell proliferation, apoptosis, and metastasis [13, 14]. A study by Xu et al. has reported that BASP1 could suppress WT1 and further inhibit the activation of the Wnt/*β*-catenin pathway in lung cancer [10]. However, whether BASP1 affects GC through regulating the Wnt/*β*-catenin pathway is unknown.

In our study, we investigated the function and molecular mechanism of BASP1 on GC. Our results demonstrated that BASP1 could suppress cell proliferation, migration, and invasion, as well as facilitate apoptosis through inhibiting the activation of Wnt/*β*-catenin pathway in GC. The findings of our study may provide a new theoretical foundation for exploring novel treatments for GC.

## 2. Materials and Methods

### 2.1. Human Tissue Samples

GC tissue samples and their adjacent noncancerous tissues were collected from 30 patients who underwent surgical resection at our hospital from July 2018 to June 2019. None of the patients before this study had received chemotherapy or radiotherapy. Written informed consent was signed by all recruited patients, and the study protocol was approved by the Ethics Committee of Yucheng People's Hospital of Shandong Province.

### 2.2. Cell Cultures

The human normal gastric epithelial cell line (GES-1) and gastric cancer cell lines (MKN-45, SNU-1, AGS, and HGC-27) were supplied by the Type Culture Collection of the Chinese Academy of Sciences (Shanghai, China). All the cells were cultured in RPMI-1640 (Sigma, USA) with 10% fetal bovine serum (FBS, Gibco, USA), 100 *μ*g/ml penicillin (HyClone, USA) and 100 *μ*g/ml streptomycin (HyClone) at 37°C in an incubator filled with 5% CO_2_ atmosphere.

### 2.3. Cell Transfection

When AGS and HGC-27 cells were passaged at 80% confluence, the cells were seeded into 6-well plates. The pcDNA3.1-BASP1 and the empty pcDNA3.1 vectors were transfected into GC cells using Lipofectamine® 3000 Reagent (Invitrogen, USA) according to the manufacturer's protocol. The transfected AGS and HGC-27 cells were randomly divided into three groups: Control group (no treatment), pcDNA3.1-NC group (transfected with empty pcDNA3.1 vector), and pcDNA-BASP1 group (transfected with pcDNA3.1-BASP1). The pcDNA3.1-BASP1 and empty pcDNA3.1 vectors were obtained from Sangon (Shanghai, China).

### 2.4. Colony Formation Assay

Colony formation assay was performed using previous described methods [15]. After transfection, a total of 1 × 10^3^ AGS and HGC-27 cells were seeded in 6-well plates and incubated for 2 weeks. Subsequently, the cell colonies were washed with phosphate-buffered saline (PBS) three times, fixed with 4% paraformaldehyde and stained with 0.1% crystal violet solution (Sigma). The number of stained colonies was observed using a light microscope.

### 2.5. 5-Ethynyl-20-Deoxyuridine (EDU) Proliferation Assay

The proliferation of AGS and HGC-27 cells was measured as described previously by using an EDU assay kit (RiboBio, Guangzhou, China). In brief, the transfected AGS and HGC-27 cells were incubated with 50 *μ*m EDU for 2 h. And the cell nuclei were dyed with 4′,6-diamidino-2phenylindole (DAPI, Sigma, USA). Finally, the EDU-positive cells were observed using a fluorescence microscope.

### 2.6. Cell Apoptosis Assay

The apoptosis of AGS and HGC-27 cells was assessed by using an Annexin V-fluorescein isothiocyanate/propidium iodide (FITC/PI) apoptosis detection kit (Invitrogen, USA), according to the manufacturer's instructions. Simply, the transfected AGS and HGC-27 cells were harvested and resuspended in binding buffer. Next, Annexin V-FITC and PI were added to the cell suspension to stain the AGS and HGC-27 cells. At the end, the apoptotic cells were examined with a flow cytometer (BD Biosciences, USA).

### 2.7. Transwell Assay

The invasion and migration abilities of AGS and HGC-27 cells were measured by using a Transwell Boyden chamber (8 *μ*m pore inserts; Corning, Corning, USA) coated with or without Matrigel (Millipore, Billerica, USA). After transfection, AGS and HGC-27 cells were resuspended in serum-free medium containing 1% bovine serum albumin (BSA, Gibco) and planted into the upper chamber. The lower chamber was filled with 500 *μ*l of medium containing 10% FBS. After culturing for 24 h, the cells on the upper surface were carefully wiped away. And the migrated or invaded cells on the underside of the membrane were fixed for 15 min at room temperature with 4% paraformaldehyde and stained with 0.1% crystal violet (Sigma, USA) for 15 min at room temperature. Finally, the number of migrated and invaded cells was counted using an optical microscope.

### 2.8. Quantitative Real-Time Polymerase Chain Reaction (qRT-PCR)

Total RNA from GC tissue and GC cells was extracted using the TRIzol kit (Invitrogen, USA) following the manufacturer's protocols. Total RNA was then reverse-transcribed into the complementary deoxyribose nucleic acid (cDNA) using the revert aid first-strand cDNA synthesis kit (Thermo Fisher, USA). qRT-PCR was performed using the SYBR Green One-Step RT-PCR Master Mix (Thermo Fisher, USA) on a 7900 real-time PCR system (Applied Biosystems, USA). The primer sequences were designed by Sangon and listed as follows: BASP1 (sense): 5′-CTTCAGACT CAAAACCCGGC-3′, (antisense): 5′-ACGGTTTGGTCGGAATTAGC-3′; WT1 (sense): 5′-CACAGCACAGGGTACGAGAG-3′, (antisense): 5′-CAAGAGTCGGGGCTACTCCA-3′; Wnt (sense): 5′-ACGCTGACTTCGGCGTGTTAG-3′, (antisense): 5′-CGTGGCACTTGCATTTGAGG-3′; *β*-catenin (sense): 5′-ATTTGATGGAGTTGGACATGGC-3′, (antisense): 5′-GAGGAAGAGGATGTGGATACCTCC-3′; and *β*-actin (sense): 5′-AGGCACCAGGGCGTGAT-3′, (antisense): 5′-GCCCACATAGGAATCCTTCTGAC-3′.

### 2.9. Western Blot Analysis

Western blotting was performed according to standard techniques as described previously [15]. Briefly, total protein was extracted from AGS and HGC-27 cells by RIPA lysis buffer (Beyotime, Shanghai, China) and maintained at -80°C until use. Total protein was separated on 10% SDS-PAGE and then transferred onto PVDF membranes. After blocking, the treated membranes were incubated with primary antibodies (Bax, 1 : 1000, ab77566; caspase-3, 1 : 1000, ab13847; Bcl-2, 1 : 1000, ab185002; matrix metalloproteinases 2 (MMP-2), 1 : 1000, ab97779; MMP-9, 1 : 1000, ab38898; WT1, 1 : 500, ab216646; and Wnt, 1 : 1000, ab15251; and *β*-catenin, 1 : 1000, ab32572) overnight at 4°C. Subsequently, the membranes were incubated with the secondary antibody for 2 h. Finally, the protein bands were visualized with an ECL system (Amersham Biosciences, UK).

### 2.10. Statistical Analyses

Experimental data are presented as the mean ± SD. All statistical analyses were performed using GraphPad Prism 8.0 (GraphPad Software Inc.). Student's *t*-test and one-way ANOVA followed by Tukey's post hoc analysis were used for the analysis of significant differences. *P* < 0.05 was considered to indicate a statistically significant difference.

## 3. Results

### 3.1. The Expression of BASP1 Is Downregulated in GC

**Figure 1:**
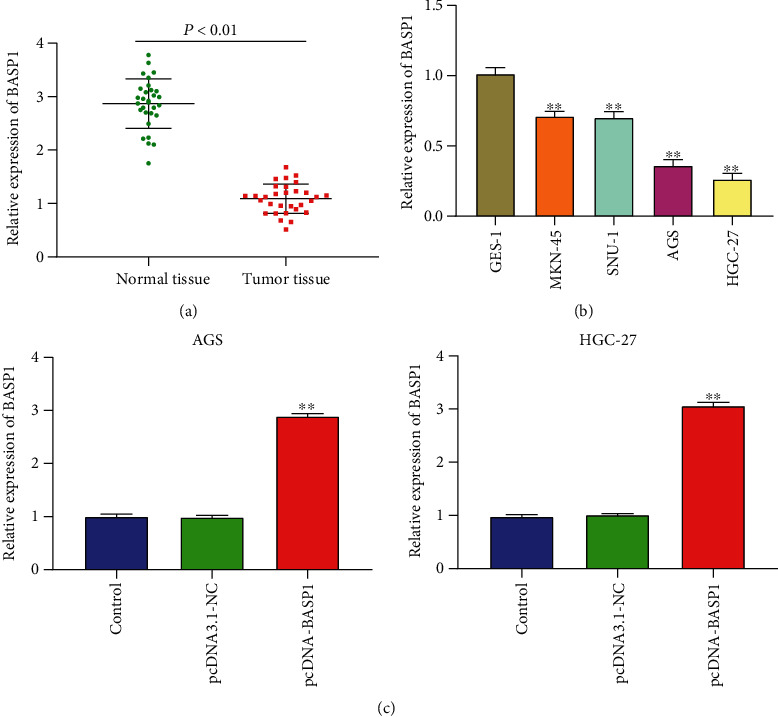
The expression of BASP1 was downregulated in GC. (a) The qRT-PCR results demonstrated that BASP1 expression in GC tissue was significantly lower than that in adjacent normal tissue. (b) The qRT-PCR results also demonstrated that BASP1 expression was markedly decreased in several GC cell lines (MKN-45, SNU-1, AGS, and HGC-27) compared with normal gastric epithelium cell line (GES-1). (c) AGC and HGC-27 cells were both transfected with pcDNA-BASP1. Transfection efficiency was analyzed with qRT-PCR. ∗∗*P* < 0.01 vs. the normal tissue group (a); ∗∗*P* < 0.01 vs. GES-1 cells group (b); ∗∗*P* < 0.01 vs. the Control and pcDNA3.1-NC groups (c).

The results of qRT-PCR showed that the expression of BASP1 in GC tissue was significantly lower than that in normal gastric tissues (*P* < 0.01) (Figure 1(a)). Similarly, compared with GES-1 cells, the expression of BASP1 was dramatically decreased in MKN-45 and SNU-1 cells, especially in AGS and HGC-27 cells (*P* < 0.01) (Figure 1(b)). Thus, we selected AGS and HGC-27 cells for subsequent experiments. As shown in Figure 1(c), BASP1 expression in AGS and HGC-27 cells was markedly increased in the pcDNA-BASP1 group when compared with the Control and pcDNA3.1-NC groups (*P* < 0.01) (Figure 1(c)), suggesting that the transfection was successful. All the results indicated that BASP1 was lowly expressed in GC.

### 3.2. BASP1 Overexpression Inhibits the Proliferation of AGS and HGC-27 Cells

**Figure 2:**
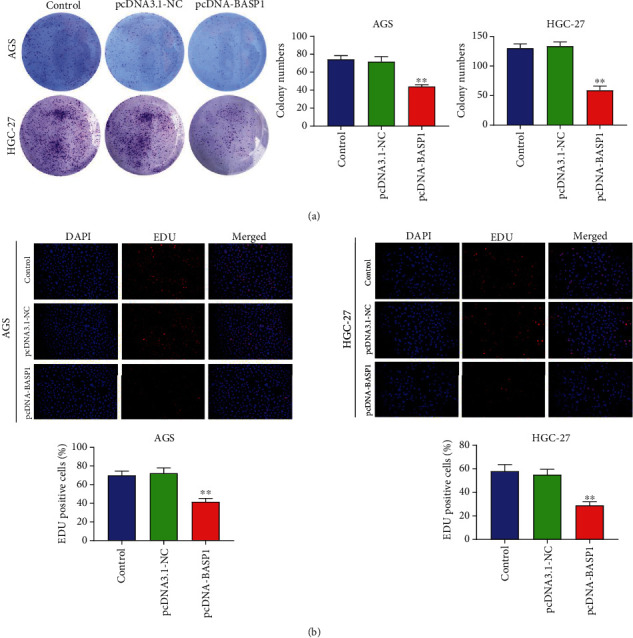
BASP1 overexpression inhibited the proliferation of AGS and HGC-27 cells. (a) Colony formation assay demonstrated that BASP1 overexpression significantly inhibited the colony number of AGS and HGC-27 cells. (b) EDU assay revealed that BASP1 overexpression significantly suppressed the proliferation of AGS and HGC-27 cells. ∗∗*P* < 0.01 vs. the Control and pcDNA3.1-NC groups.

The proliferation potential of AGS and HGC-27 cells was assessed by the colony formation assay and the EDU assay.  Figure 2(a) results show that BASP1 overexpression markedly suppressed the clone numbers of AGS and HGC-27 cells relative to the Control and pcDNA3.1-NC groups (*P* < 0.01). In addition, the EDU assay results also confirmed that BASP1 overexpression significantly inhibited the proliferation of AGS and HGC-27 cells (*P* < 0.01) (Figure 2(b)). All data revealed that BASP1 overexpression could inhibit the proliferation of AGS and HGC-27 cells.

### 3.3. BASP1 Overexpression Promotes the Apoptosis of AGS and HGC-27 Cells

**Figure 3:**
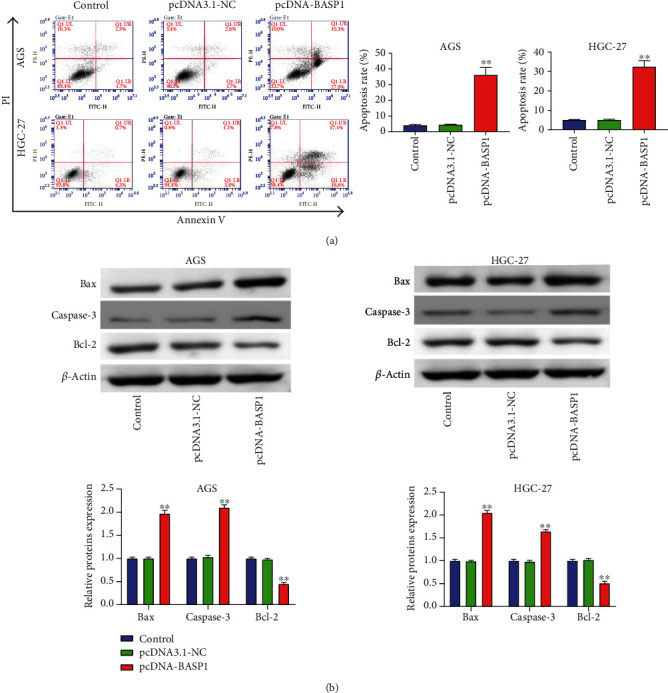
BASP1 overexpression promoted the apoptosis of AGS and HGC-27 cells. (a) Flow cytometry analysis showed that the transfection of pcDNA-BASP1 markedly promoted the apoptosis of AGS and HGC-27 cells. (b) Western blot analysis revealed that BASP1 overexpression significantly increased the expression of Bax and caspase-3 in AGS and HGC-27 cells, but notably decreased Bcl-2 expression. ∗∗*P* < 0.01 vs. the Control and pcDNA3.1-NC groups.

The results of flow cytometry showed that BASP1 overexpression significantly increased apoptosis in AGS and HGC-27 cells compared with the Control and pcDNA3.1-NC groups (*P* < 0.01) (Figure 3(a)). To confirm the role of BASP1 in GC cell apoptosis, we further measured the expression of apoptosis-related proteins (Bax, caspase-3, and Bcl-2) by western blot. The results revealed that BASP1 overexpression significantly elevated the expression of Bax and caspase-3 in AGS and HGC-27 cells (*P* < 0.01) but reduced the expression level of Bcl-2 (*P* < 0.01) (Figure 3(b)). These results suggested that BASP1 overexpression could facilitate the apoptosis of AGS and HGC-27 cells.

### 3.4. BASP1 Overexpression Inhibits the Migration and Invasion in AGS and HGC-27 Cells

**Figure 4:**
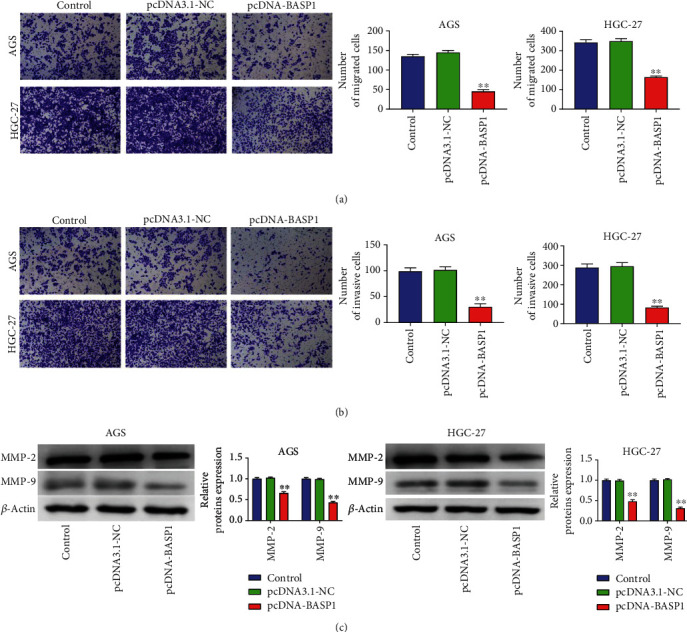
BASP1 overexpression inhibited the migration and invasion in AGS and HGC-27 cells. (a) Transwell assay showed that BASP1 overexpression significantly suppressed the migration of AGS and HGC-27 cells. (b) Transwell assay also revealed that BASP1 overexpression markedly inhibited the invasion of AGS and HGC-27 cells. (c) Western blot analysis revealed that BASP1 overexpression significantly reduced the expression of MMP-2 and MMP-9 in AGS and HGC-27 cells. ∗∗*P* < 0.01 vs. the Control and pcDNA3.1-NC groups.

As shown in Figure 4(a), BASP1 overexpression markedly reduced the migration of AGS and HGC-27 cells when compared with the Control and pcDNA3.1-NC groups (*P* < 0.01) (Figure 4(a)). Similarly, BASP1 overexpression significantly also repressed the invasion of AGS and HGC-27 cells relative to the Control and pcDNA3.1-NC groups (*P* < 0.01) (Figure 4(b)). To confirm the function of BASP1 on GC cell migration and invasion, we further measured the expression of MMP-2 and MMP-9 by western blot. The results showed that the expression of MMP-2 and MMP-9 in AGS and HGC-27 cells was dramatically reduced by BASP1 overexpression (*P* < 0.01) (Figure 4(c)). All the data indicated that BASP1 overexpression could inhibit the migration and invasion of AGS and HGC-27 cells.

### 3.5. BASP1 Overexpression Inhibits the Activation of the Wnt/*β*-Catenin Pathway in AGS and HGC-27 Cells

**Figure 5:**
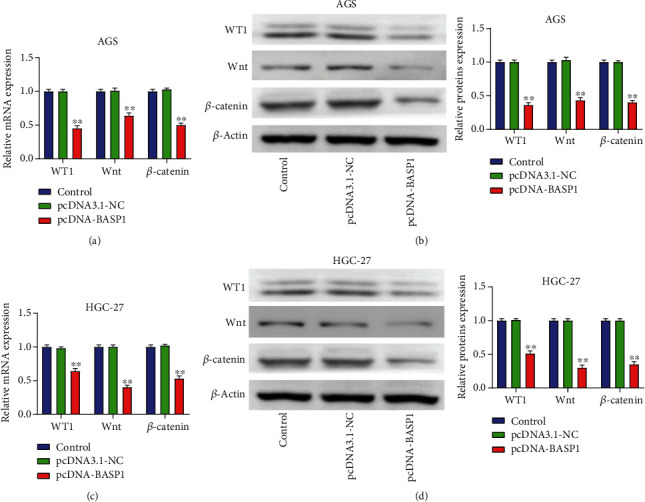
BASP1 overexpression inhibited the activation of the Wnt/*β*-catenin pathway in AGS and HGC-27 cells. (a) The qRT-PCR results showed that BASP1 overexpression significantly decreased the mRNA expressions of WT1, Wnt, and *β*-catenin in AGS cells. (b) Western blot analysis also revealed that BASP1 overexpression markedly reduced the protein expressions of WT1, Wnt, and *β*-catenin in AGS cells. (c) The qRT-PCR results revealed that BASP1 overexpression significantly decreased the mRNA expressions of WT1, Wnt, and *β*-catenin in HGC-27 cells. (d) Western blot analysis also demonstrated that BASP1 overexpression significantly decreased the protein expressions of WT1, Wnt, and *β*-catenin in HGC-27 cells. ∗∗*P* < 0.01 vs. the Control and pcDNA3.1-NC groups.

As shown in Figure 5(a), the mRNA and protein expressions of WT1, Wnt, and *β*-catenin in AGS cells were dramatically decreased in pcDNA-BASP1 group relative to the Control and pcDNA3.1-NC groups (*P* < 0.01). Similarly, when compared with the Control and pcDNA3.1-NC groups, the mRNA and protein expressions of WT1, Wnt, and *β*-catenin in HGC-27 cells were also reduced in the pcDNA-BASP1 group (*P* < 0.01) (Figure 5(b)). All the results suggested that BASP1 overexpression could inhibit the activation of the Wnt/*β*-catenin pathway in AGS and HGC-27 cells.

## 4. Discussion

GC is a global health-threatening malignancy with more than 1,000,000 new cases diagnosed each year [16]. In China, more than 320,000 fatalities due to GC are reported each year [17]. In addition, the current treatment for GC has gradually improved in the past few decades, but the prognosis of GC patients remains poor. Therefore, it is important to explore the relevant molecular biomarkers and mechanisms for GC at the current stage. In the present study, we demonstrated that BASP1 could suppress cell proliferation, migration, and invasion, as well as facilitate apoptosis through inhibiting the activation of the Wnt/*β*-catenin pathway in GC.

BASP1 is identified as a tumor suppressor and is lowly expressed in multiple cancers. For example, the expression of BASP1 is reported to be downregulated in thyroid cancer tissues [5]. A study by Zhou et al. has demonstrated that BASP1 expression is also downregulated in acute myeloid leukemia [18]. In this study, we found that BASP1 expression in GC tissue and cells was significantly lower than that in normal tissue and cells, which was in line with previous studies and suggested that BASP1 might be a tumor suppressor of GC. In addition, a growing body of evidence has reported that BASP1 could modulate a variety of tumor cell biological behaviors. For instance, BASP1 is confirmed to repress the growth of acute myeloid leukemia by inhibiting cell proliferation and promoting cell apoptosis [18]. Guo et al. [5] have suggested that BASP1 could inhibit cell proliferation and migration, as well as promote cell apoptosis in thyroid cancer. In the present study, we confirmed that BASP1 overexpression markedly suppressed cell proliferation, migration, and invasion and facilitated cell apoptosis in GC cells. Previous studies have indicated that BASP1 could regulate many genes related to apoptosis, such as Bax and c-Myc [8, 11]. Our data showed that BASP1 overexpression could facilitate GC cell apoptosis by regulating the expression of Bax, caspase-3, and Bcl-2. Moreover, we confirmed that BASP1 overexpression could inhibit GC cell migration and invasion by modulating the expression of MMP-2 and MMP-9.

In recent years, WT1 has been reported as an oncogene regulating the progression of malignancies [19]. Carpenter et al. [11] have confirmed that BASP1 could inhibit the transcriptional activation of WT1. Moreover, it has been documented that WT1 can activate the Wnt/*β*-catenin pathway and facilitate cell migration and invasion in tumors [10]. The Wnt/*β*-catenin pathway is highly conserved and can regulate a series of biological processes in the progression of diseases [20, 21]. In addition, an increasing number of studies have demonstrated that the Wnt/*β*-catenin pathway plays an important role in multiple cancers, including GC [22–24]. In addition, the Wnt/*β*-catenin pathway has been reported to regulate cell biological behaviors including cell proliferation, apoptosis, and metastasis [25]. Chen et al. [26] have reported that TYRO3 promotes cell growth and metastasis through activating the Wnt/*β*-catenin pathway in GC. Zhong et al. [27] have suggested that LOC285194 could facilitate GC progression through the activation of the Wnt/*β*-catenin pathway. In the present study, we explored the effect of BASP1 on the expression of WT1, Wnt, and *β*-catenin in GC cells by qRT-PCR and western blot assays. The results confirmed that BASP1 overexpression could significantly inhibit the expression of WT1, Wnt, and *β*-catenin, indicating that BASP1 overexpression could inhibit the activation of Wnt/*β*-catenin pathway in GC. However, our study has some limitations, such as the absence of *in vivo* experiments. The function of BASP1 in *in vivo* experiments will be explored further in our future studies.

In summary, our work revealed that the expression of BASP1 was downregulated in both GC tissues and cells. We also demonstrated that BASP1 could significantly suppress cell proliferation, migration, and invasion and promote apoptosis through inhibiting the activation of the Wnt/*β*-catenin pathway in GC. These findings suggest that BASP1 may serve as a therapeutic target for the treatment of GC in the future.

## Data Availability

The datasets used and analyzed during the current study are available from the corresponding author on reasonable request.
